# Brentuximab vedotin plus CHP-like versus CHOEP regimen for the first-line treatment of PTCL patients: a multi-center propensity-matched retrospective study

**DOI:** 10.3389/fimmu.2026.1973646

**Published:** 2026-10-21

**Authors:** Jing Chen, Fangshu Guan, Zhipeng Cheng, Yingying Wu, Lin Liu, Lu Zhang, Fang Liu, Guohui Cui, Heng Mei, Fanjun Cheng, Yi Zhao, Jun Fang

**Affiliations:** 1 Institute of Hematology, Union Hospital, Tongji Medical College, Huazhong University of Science and Technology, Wuhan, Hubei, China; 2 Bone Marrow Transplantation Center, The First Affiliated Hospital, School of Medicine, Zhejiang University, Hangzhou, Zhejiang, China; 3 Department of Hematology, Yichang National Regional Medical Center, Yichang, Hubei, China

**Keywords:** brentuximab vedotin, CHOEP, CHP-like, first-line treatment, outcome, peripheral T-cell lymphoma

## Abstract

**Introduction:**

The optimal frontline treatment option for Peripheral T-cell lymphomas (PTCLs) has not been completely defined.

**Methods:**

To compare the efficacy and safety of brentuximab vedotin (BV) combined with CHP-like regimen (cyclophosphamide, an anthracycline component, and prednisone) and CHOEP regimen (cyclophosphamide, doxorubicin, vincristine, prednisone and etoposide) as first-line treatment for newly diagnosed PTCLs, we used 1:1 optimal propensity score-matching (PSM) to match patients by gender, age, disease stage, bone marrow infiltration, extranodal involvement, Eastern Cooperative Oncology Group performance status, and PTCL subtype, resulting in two cohorts of 45 patients each.

**Results:**

With a median follow-up of 27.2 months, the 2-year progression-free survival (PFS) rates were 70.7% (95% confidence interval [CI]: 58.5%-85.5%) and 45.3% (95% CI: 32.7%-62.7%) for the BV plus CHP-like and CHOEP groups, respectively (P = 0.012). The 2-year overall survival (OS) rate was 81.0% (95% CI: 68.8%-95.3%) in the BV plus CHP-like group and 75.1% (95% CI: 62.7%-90.1%) in the CHOEP group (P = 0.550). No significant differences were observed between the two groups in complete remission (CR, P = 0.673) and objective response rate (ORR, P = 0.662) at the end-of-follow-up assessment. The duration of response (DOR) in the BV plus CHP-like group was significantly prolonged compared to the CHOEP group (81.9% vs. 63.0%, P = 0.035). In subgroup analysis stratified by ALK expression, no deaths occurred among the 31 ALK-positive ALCL patients; the 2-year PFS rate was 94.4% versus 76.9% (P = 0.066). Among the 59 non-ALK-positive PTCL patients, no significant differences were observed in OS (P = 0.880) or PFS (P = 0.110). BV plus CHP-like regimen was associated with a lower incidence of hematological toxicities than the CHOEP regimen, both for any-grade and for grade ≥ 3 events.

**Conclusion:**

The BV plus CHP-like regimen may improve the PFS, without increasing hematological adverse events. However, this benefit was largely offset in non-ALK-positive PTCL patients, and further validation in larger cohorts is warranted.

## Introduction

Peripheral T-cell lymphomas (PTCLs) constitute a heterogeneous subgroup of non-Hodgkin lymphomas (NHLs) that arise from mature T lymphocytes and natural killer (NK) cells, accounting for approximately 10-15% of all NHL cases globally (1, 2). PTCLs are characterized by substantial clinical heterogeneity, aggressive biological behavior, drug resistance, and unfavorable long-term prognosis (3–5). The treatment strategy for newly diagnosed PTCL is undergoing continuous refinement, and therapeutic approaches are evolving. Cyclophosphamide, doxorubicin, vincristine, and prednisone (CHOP), or cyclophosphamide, doxorubicin, vincristine, etoposide, and prednisone (CHOEP), or dose-adjusted etoposide, prednisone, vincristine, cyclophosphamide, doxorubicin (DA-EPOCH), or brentuximab vedotin (BV) plus cyclophosphamide, doxorubicin, and prednisone (CHP), are commonly recommended (6, 7). However, the optimal frontline treatment option has not been completely defined (8).

Although the CHOEP regimen has yielded conflicting results across different PTCL populations, subtype-specific evidence suggests that its benefit is most pronounced in eligible younger, fit patients with PTCL, especially those with ALK-positive anaplastic large cell lymphoma (ALCL) (9–11). In a pooled analysis of 320 patients with PTCL from seven prospective German trials, CHOEP improved event-free survival (EFS) in younger patients with normal lactate dehydrogenase (LDH) (75.4% vs 51.0%, P = 0.003), particularly in those with ALK-positive ALCL (91.2% vs 57.1%, P = 0.012), although this benefit was offset by increased toxicity in elderly patients (9). The Swedish Lymphoma Registry similarly reported that CHOEP was associated with better PFS in younger PTCL patients (HR, 0.49; 95% CI, 0.29–0.83; P = 0.008) (12). A Nordic Lymphoma Group study further found that adding etoposide to CHOP in ALK-positive ALCL seems reasonable for patients aged 41–65 years (13). Janikova et al. compared CHOP (n = 113) with CHOEP (n = 68) induction and found that CHOEP was associated with better 5-year PFS (25.0% vs 32.9%; P < 0.001) and 5-year OS (47.6% vs 65.6%; P = 0.008) (14).

BV, an antibody-drug conjugate targeting CD30, delivers cytotoxic payloads specifically to CD30-positive cells, enhancing anti-tumor efficacy while reducing off-target effects (15). According to a double-blind, double-dummy, randomized, placebo-controlled and phase III study, the BV plus CHP group showed meaningful improvement in progression-free survival (PFS) and overall survival (OS) compared with CHOP group (5-year PFS: 51.4% vs. 43.0%, P = 0.008; 5-year OS: 70.1% vs. 61.0%, P = 0.042) among untreated PTCL patients (16). As a result, the BV plus CHP regimen has emerged as a promising targeted therapeutic option for diverse PTCL subtypes, especially CD30-positive PTCL (≥10% CD30 expression) (17, 18). Although BV plus CHP has been observed to achieve better therapeutic outcomes compared with CHOP, the efficacy comparisons between BV plus CHP and CHOEP are still insufficient in existing clinical investigations.

In this two-center retrospective study, we aimed to evaluate the efficacy and safety of the BV-based CHP regimen in the first-line treatment of untreated PTCL patients by real-world data. Specifically, the BV plus CHP regimen was compared with the standard CHOEP regimen in terms of therapeutic efficacy, long-term survival outcomes, and toxic profile with propensity-score matched (PSM) method.

## Materials and methods

### Patient enrollment and data collection

This was a multi-center, retrospective cohort study conducted across two large tertiary hospitals in China between January 2014 and December 2024. A total of 113 eligible untreated PTCL patients were enrolled according to inclusion and exclusion criteria (**Supplementary Figure 1**). PSM was performed using a 1:1 optimal matching approach, with matching variables including gender, age, disease stage, bone marrow infiltration (BMI), extranodal involvement, Eastern Cooperative Oncology Group performance status (ECOG PS), and PTCL subtypes. After matching, two cohorts of 45 patients each were generated for the BV plus CHP-like and CHOEP groups, respectively. The International Prognostic Index (IPI) and Prognostic Index for PTCL (PIT) (19) were used for prognostic evaluation.

### Treatment

Patients received intravenous BV at a dose of 1.8 mg/kg on day 1 of each 21-day cycle. The CHP-like regimen comprised cyclophosphamide (C, 750 mg/m^2^), an anthracycline component (H: doxorubicin at 50 mg/m^2^, or liposomal doxorubicin at 30 mg/m^2^, or epirubicin at 70 mg/m^2^) administered intravenously on day 1, and prednisone (P, 100 mg) administered orally on days 1–5 each 21-day cycle. The CHOEP regimen included cyclophosphamide (750 mg/m^2^ intravenously on day 1), doxorubicin (50 mg/m^2^ intravenously on day 1), vincristine (1.4 mg/m^2^, maximum dose 2 mg) or vindesine (4 mg on day 1), prednisone (100 mg orally on days 1-5), and etoposide (100 mg/m^2^ intravenously on days 1-3).

### Assessment of efficacy and adverse events

Imaging modalities used for efficacy assessment included 18F-FDG positron emission tomography-computed tomography (PET-CT) and computed tomography (CT). For patients with evidence of bone marrow or gastrointestinal involvement, bone marrow aspiration and biopsy, as well as endoscopic biopsy, were performed respectively to optimize diagnostic evaluation. Lymphoma treatment responses were classified in accordance with standard criteria, encompassing objective response rate (ORR) — defined as the composite of complete remission (CR) and partial remission (PR) — as well as stable disease (SD) and progressive disease (PD), all of which were assessed in strict adherence to the 2014 Lugano Classification for Lymphomas (20). The primary endpoint of this study was PFS. Key secondary endpoints were OS, ORR, CR, duration of response (DOR), and safety. Adverse events (AEs) grading were graded in accordance with the Common Terminology Criteria for Adverse Events (CTCAE) Version 5.0. More details are available in **Supplementary Materials**.

### Statistical analysis

Differences between categorical variables were assessed by the χ^2^ test or Fisher’s exact test. Differences between continuous variables were assessed by the Mann-Whitney U test. OS and PFS were analyzed using Kaplan-Meier method. All data were analyzed using R version 4.2.2 or SPSS version 27. Ninety-five percent confidence interval (CI) was calculated to assess the precision of estimates. P values were two-sided, and the value of P < 0.05 indicated a statistically significant difference.

## Results

### Baseline characteristics among matched cohort

After PSM (1:1 optimal matching), a total of 90 patients were included in the analysis, with 45 patients in the BV plus CHP-like group and 45 patients in the CHOEP group. All baseline characteristics of the patients in the two groups are summarized in **Table 1**. Both groups had about 3/4 of patients with Ann Arbor stage III/IV disease (P = 1.000). Patients with high-intermediate or high risk by IPI (IPI = 3-5) accounted for approximately 30% in each group. The distribution of disease subtypes did not differ significantly between the two groups (P = 0.307). The proportion of patients who underwent hematopoietic stem cell transplantation (HSCT) after first-line treatment was identical (13.3%, P = 1.000). The full pre- and post-matching standardized mean differences (SMDs) for all covariates are shown in **Supplementary Table 1**, and the corresponding Love plot are presented in **Supplementary Figure 2**. After PSM, the two groups were comparable for most baseline characteristics, although residual imbalance remained for LDH, extranodal involvement, and pathological subtype (SMD > 0.2). These imbalanced factors were further evaluated in sensitivity analyses to assess their potential impact on the study outcomes, and more details are shown in the **Supplementary Materials**.

**Table 1:** Baseline clinical characteristics of matched patients with peripheral T-cell lymphoma.

Characteristics	BV plus CHP-like group (n=45)	CHOEP group (n= 45)	P value
Male, no. (%)	28 (62.2)	28 (62.2)	1.000
Age >60 years, no. (%)	15 (33.3)	12 (26.7)	0.646
Ann Arbor stage III/IV, (n, %)	34 (75.6)	35 (77.8)	1.000
ENSs ≥2, no. (%)	14 (31.1)	9 (20.0)	0.334
BMI, no. (%)	10 (22.2)	11 (24.4)	1.000
ECOG PS ≥2, no. (%)	5 (11.1)	3 (6.7)	0.714
Elevated LDH, no. (%)	16 (35.6)	25 (55.6)	0.090
Albumin [median (range), g/L]	38.8 (19.5-50.8)	38.0 (1.2-49.1)	0.753
WBC [median (range), ×10^9^/L]	8.1 (2.8-40.1)	6.4 (2.4-82.0)	0.470
HB [median (range), g/L]	127.0 (75-157)	124 (66-167)	0.753
PLT [median (range), ×10^9^/L]	232 (21-496)	199 (18-458)	0.625
IPI, no. (%)			0.973
0-1 (a Low IPI)	16 (35.6)	17 (37.8)	
2 (a Low-Intermediate IPI)	15 (33.3)	15 (33.3)	
3 (a High-Intermediate IPI)	11 (24.4)	11 (24.4)	
4-5 (a High IPI)	3 (6.7)	2 (4.4)	
PIT, no. (%)			0.645
Group 1: 0 risk factor	14 (31.1)	13 (28.9)	
Group 2: 1 risk factor	19 (42.2)	20 (44.4)	
Group 3: 2 risk factors	9 (20.0)	6 (13.3)	
Group 4: 3–4 risk factors	3 (6.7)	6 (13.3)	
Subtype, no. (%)			0.307*
AITL	10 (22.2)	15 (33.3)	
PTCL-TFH	3 (6.7)	2 (4.4)	
PTCL NOS	9 (20.0)	13 (28.9)	
ALCL	23 (51.1)	15 (33.3)	
ALK positive	18 (40.0)	13 (28.9)	
ALK negative	5 (11.1)	2 (4.4)	
Transplantation, no. (%)	6 (13.3)	6 (13.3)	1.000

*The P value for subtype refers to the overall distribution across the four PTCL subtype categories (AITL, PTCL-TFH, PTCL-NOS, and ALCL).

ENSs, extranodal sites; BMI, bone marrow infiltration; ECOG PS, Eastern Cooperative Oncology Group performance status; LDH, lactate dehydrogenase; WBC, white blood cell; HB, hemoglobin; PLT, platelet count; IPI, International Prognostic Index; PIT, prognostic index for PTCL; AITL, angioimmunoblastic T-cell lymphoma; PTCL-TFH, nodal T-follicular helper (TFH) cell lymphoma; PTCL NOS, peripheral T-cell lymphoma, not otherwise specified; ALCL, anaplastic large cell lymphoma.

### Efficacy

For the entire cohort, the 2-year OS was 78.0% (95% CI: 69.0%-88.1%), while 2-year PFS was 57.9% (95% CI: 48.5%-69.3%) with a median follow-up of 27.2 (95% CI: 22.6-34.0) months. Median follow-up was 25.8 (95% CI: 17.3-32.7) months in the BV plus CHP-like group and 33.0 (95% CI: 23.2-44.2) months in the CHOEP group. After matching, no significant difference in OS was observed between the BV plus CHP-like and CHOEP groups (P = 0.550). The median OS was not reached in either group. The 2-year OS rate was 81.0% (95% CI: 68.8%-95.3%) in the BV plus CHP-like group and 75.1% (95% CI: 62.7%-90.1%) in the CHOEP group (P = 0.550, **Figure 1A**). In contrast, PFS showed a statistically significant difference (P = 0.012). The median PFS was not reached in the BV plus CHP-like group, whereas it was 11.4 months in the CHOEP group. The 2-year PFS rates were 70.7% (95% CI: 58.5%-85.5%) and 45.3% (95% CI: 32.7%-62.7%) for the BV plus CHP-like and CHOEP groups, respectively (P = 0.012, **Figure 1B**).

**Figure 1:**
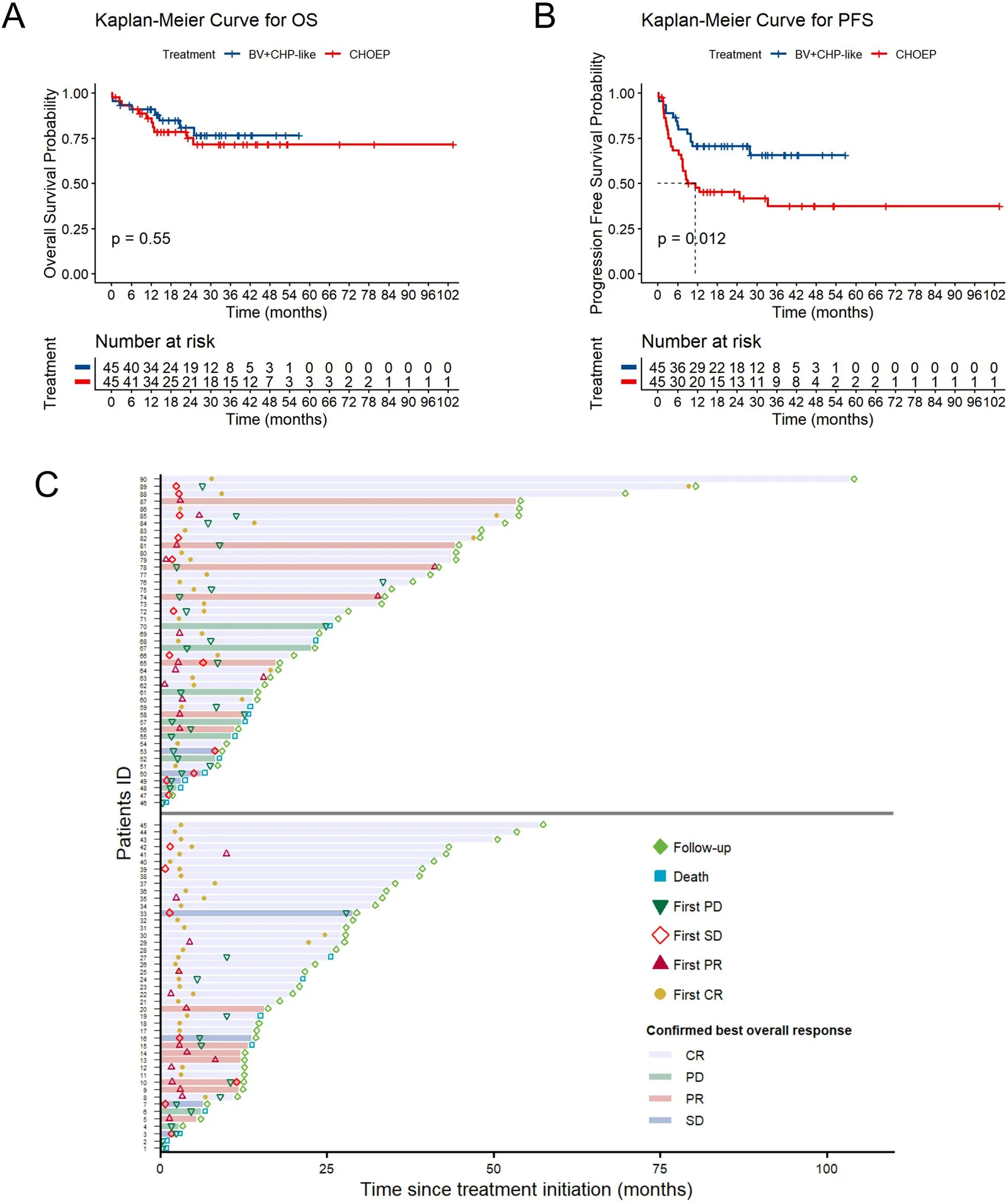
Survival analysis and clinical response profiles of BV plus CHP-like (IDs 1-45) vs. CHOEP (IDs 46-90) regimens in newly diagnosed peripheral T-cell lymphomas. **(A)** Overall survival (OS) Kaplan-Meier curve; **(B)** Progression-free survival (PFS) Kaplan-Meier curve; **(C)** swim plot of individual patient response trajectories over time. OS, overall survival; PFS, progression-free survival; CR, complete remission; PR, partial remission; SD, stable disease; PD, progressive disease.

To clearly depict the temporal differences in survival outcomes and event distribution between the two groups, a swimmer plot was constructed (**Figure 1C**). As illustrated in this plot, patients in the CHOEP group (IDs 46-90) had a longer follow-up duration compared with those in the BV plus CHP-like group (IDs 1-45). Disease progression events in the BV plus CHP-like group occurred at a later time point. This visualization illustrates individual follow-up and event patterns.

Several sensitivity analyses were performed to address potential confounding factors, including transplantation, treatment era, BV maintenance, and anthracycline type. Specifically, we excluded patients treated before 2020 (**Supplementary Figure 3**), those who received BV maintenance (**Supplementary Figure 4**), who underwent transplantation (**Supplementary Figure 5**), and those who received liposomal doxorubicin or epirubicin (**Supplementary Figure 6**). The results were consistent with the primary analysis: the BV plus CHP-like group showed significantly better PFS and DOR than the CHOEP group. Detailed results and corresponding figures are provided in the **Supplementary Materials**.

### Response

The treatment responses of the two groups are summarized in **Table 2**. All patients in the two groups were available for response assessment at the interim treatment phase. The ORR was 80.0% in the BV plus CHP-like group, which was significantly higher than that in the CHOEP group (53.3%, P = 0.013) at the interim evaluation. By the end of follow-up, 44 patients in each group were available for efficacy evaluation. The ORRs of the BV plus CHP-like and CHOEP groups were 68.2% and 61.4%, respectively, with no significant difference observed (P = 0.662, **Figure 2A**) at the end-of-follow-up assessment. No significant difference in CR rate was noted between these two regimens (56.8% vs. 50.0%, P = 0.673) at the end-of-follow-up assessment. Furthermore, the DOR was significantly prolonged in the BV plus CHP-like group compared with the CHOEP group. The 2-year DOR rates were 81.9% (95% CI: 69.6%-96.3%) and 63.0% (95% CI: 47.8%-83.0%) in the BV plus CHP-like and CHOEP groups, respectively (P = 0.035, **Figure 2B**).

**Table 2:** Response rate assessment.

Efficacy (%, n)	BV plus CHP-like group (n=45)		CHOEP group (n= 45)	
	Interim evaluation, no. (%)	End-of-follow-up evaluation, no. (%)	Interim evaluation, no. (%)	End-of-follow-up evaluation, no. (%)
Overall response	36 (80.0)	30 (68.2)	24 (53.3)	27 (61.4)
Complete response	23 (51.1)	25 (56.8)	15 (33.3)	22 (50.0)
Partial response	13 (28.9)	5 (11.4)	9 (20.0)	5 (11.4)
Stable disease	4 (8.9)	1 (2.3)	7 (15.6)	1 (2.3)
Progressive disease	5 (11.1)	13 (29.5)	14 (31.1)	16 (36.4)
Missing	0	1/45	0	1/45

Response rates at end of follow-up were calculated among patients with evaluable response data (n = 44 per group), excluding one patient with missing data in each group.

**Figure 2:**
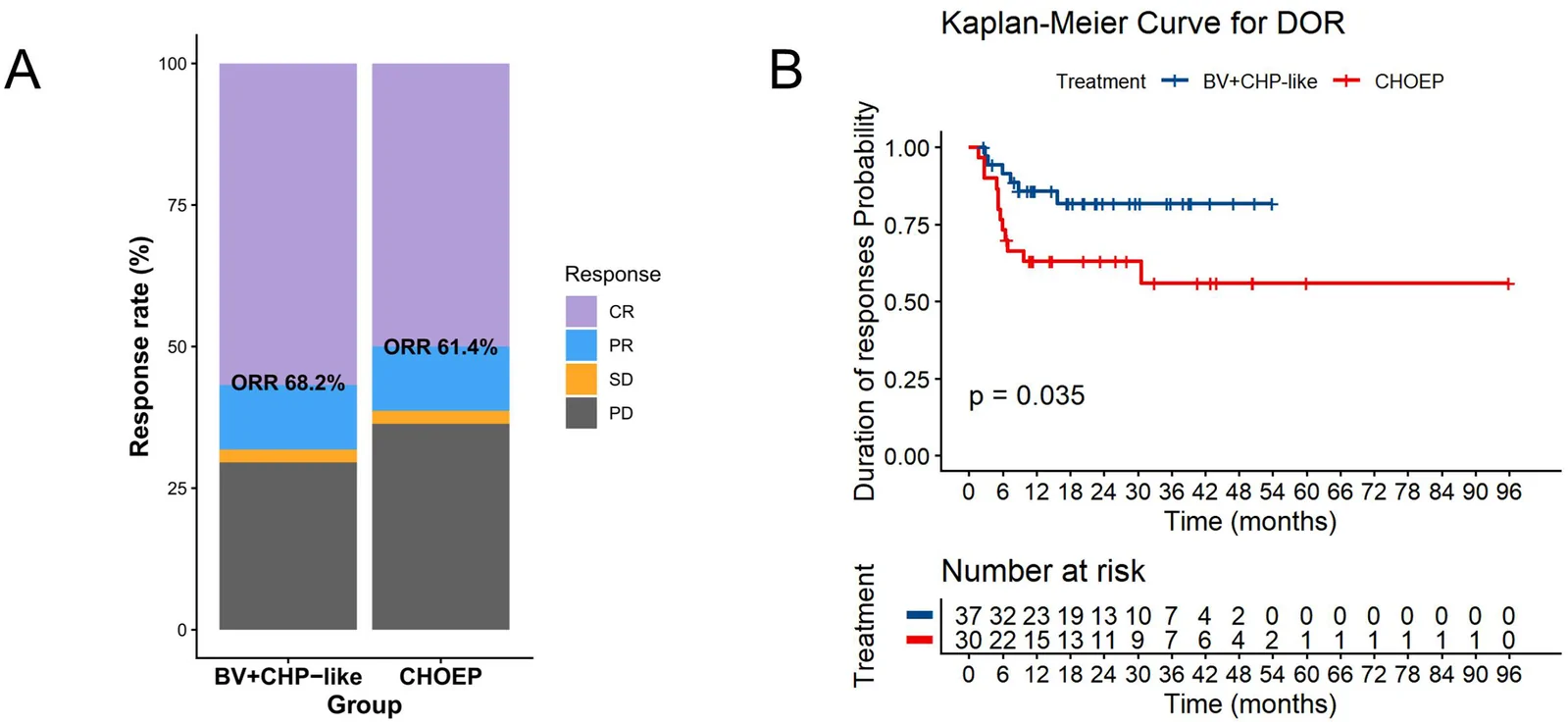
Efficacy outcomes of BV plus CHP-like vs. CHOEP regimens in newly diagnosed peripheral T-cell lymphomas at the end of follow-up assessment. **(A)** Response rate distribution; **(B)** Kaplan-Meier curve for duration of response (DOR). CR, complete remission; PR, partial remission; SD, stable disease; PD, progressive disease; ORR, objective response rate; DOR, duration of response.

### Subgroup survival analysis

Given the inherently favorable prognosis of ALK-positive ALCL, we conducted subgroup survival analyses stratified by ALK expression. Among the 31 ALK-positive ALCL patients (n = 18 in the BV plus CHP-like group and n = 13 in the CHOEP group), no deaths were observed in either group, leading to a comparable 100% OS rate (P = 1.000, **Figure 3A**). The 2-year PFS rate was 94.4% (95% CI: 84.4%-100%) in the BV plus CHP-like cohort versus 76.9% (95% CI: 57.1%-100%) in the CHOEP group, without reaching a statistically significant difference (P = 0.066, **Figure 3B**). For the 59 non-ALK-positive PTCL patients (n = 27 in the BV plus CHP-like group and n = 32 in the CHOEP group), no statistically significant intergroup differences were observed in either OS or PFS (P = 0.880 for OS, **Figure 3C**; P = 0.110 for PFS, **Figure 3D**). After exclusion of ALK-positive ALCL, the PFS difference between the two groups was no longer statistically significant, whereas the OS difference remained non-significant.

**Figure 3:**
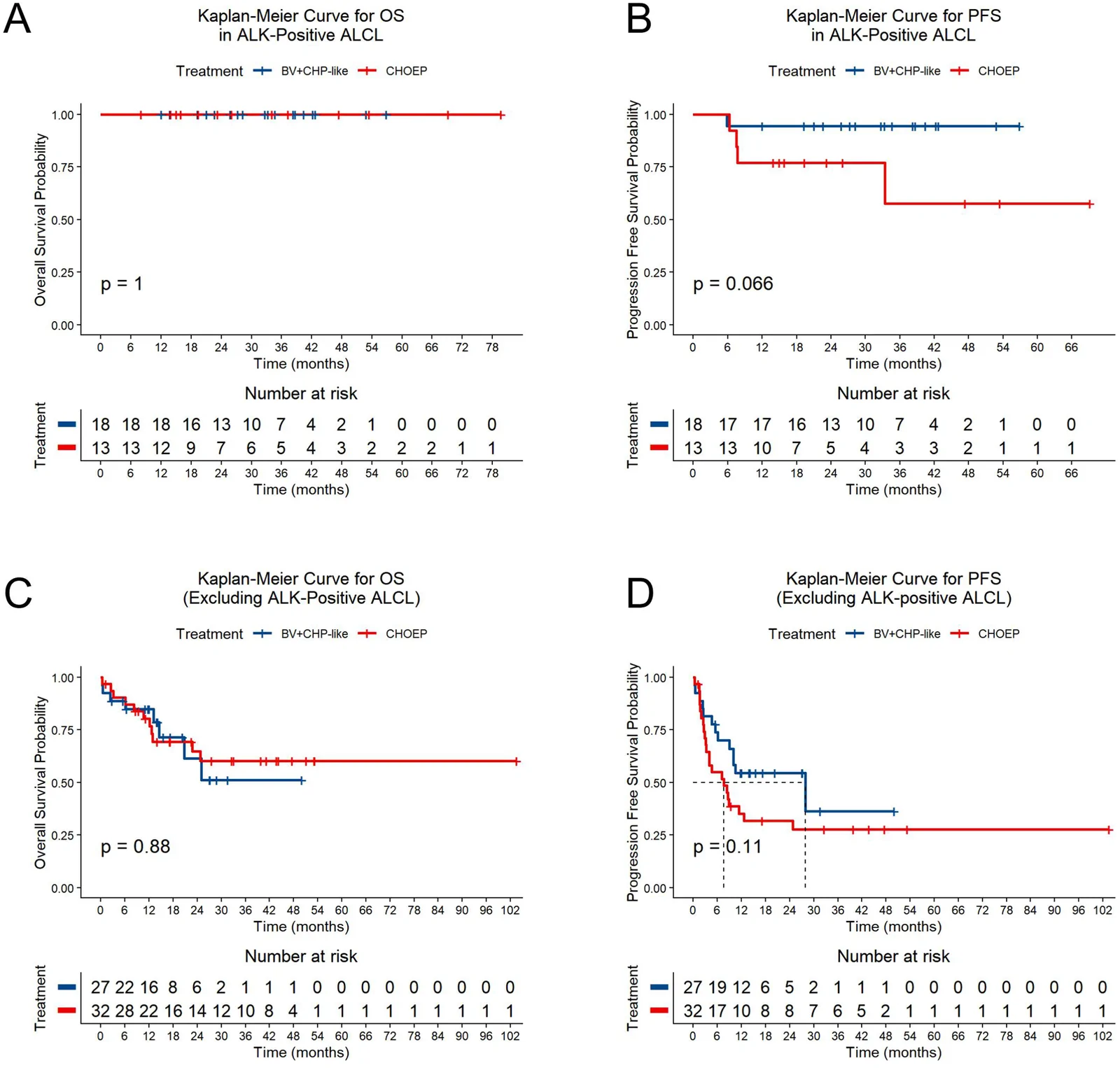
Subgroup survival analyses by ALK status. **(A, B)** Kaplan-Meier curves for overall survival (OS) and progression-free survival (PFS) in ALK-positive ALCL patients. **(C, D)** Kaplan-Meier curves for OS and PFS in non-ALK-positive PTCL patients. OS, overall survival; PFS, progression-free survival; ALCL, anaplastic large cell lymphoma.

In addition, subgroup analyses regarding study center (**Supplementary Figure 7**), the level of LDH (**Supplementary Figure 8**), and the number of extranodal involvement (**Supplementary Figure 9**) are performed in the **Supplementary Materials**. Patients with lower LDH levels or ≤1 site of extranodal involvement tended to achieve superior PFS in the BV plus CHP-like group compared with the CHOEP group. These findings warrant further validation in larger-sample cohorts.

### Safety

The whole 90 patients were available for safety assessment. Further details are shown in **Table 3**. The BV plus CHP-like group had significantly lower rates of any-grade leukopenia (P = 0.020), neutropenia (P = 0.020), anemia (P = 0.044), and thrombocytopenia (P < 0.001) compared with the CHOEP group. Similarly, there were significant differences in Grade ≥3 hematologic toxicities between the two groups. The BV plus CHP-like group had significantly lower rates of Grade ≥3 leukopenia (P < 0.001), neutropenia (P < 0.001), anemia (P < 0.001), and thrombocytopenia (P = 0.005) compared with the CHOEP group. For non-hematologic toxicities, there were no significant differences in the incidence of pneumonia (P = 0.091), nausea (P = 0.085), constipation (P = 0.748), diarrhea (P = 1.000), and peripheral neuritis (P = 0.777) between the two groups.

**Table 3:** Adverse events.

Toxicities∗	BV plus CHP-like group (n = 45)		CHOEP group (n = 45)		P value	
	Any grade, no. (%)	Grade ≥3, no. (%)	Any grade Number (%)	Grade ≥3, Number (%)	Any grade	Grade ≥3
Leukopenia	31 (68.9)	10 (22.2)	40 (88.9)	29 (64.4)	0.020	<0.001
Neutropenia	27 (60.0)	9 (20.0)	37 (82.2)	27 (60.0)	0.020	<0.001
Anemia	37 (82.2)	8 (17.8)	43 (95.6)	27 (60.0)	0.044	<0.001
Thrombocytopenia	11 (24.4)	7 (15.6)	34 (75.6)	19 (42.2)	<0.001	0.005
Pneumonia	15 (33.3)	NA	8 (17.8)	NA	0.091	NA
Nausea	22 (48.8)	NA	14 (31.1)	NA	0.085	NA
Constipation	5 (11.1)	NA	6 (13.3)	NA	0.748	NA
Diarrhea	3 (6.7)	NA	4 (8.9)	NA	1.000	NA
Peripheral Neuritis	8 (17.8)	NA	7 (15.6)	NA	0.777	NA

NA, not available; NA indicate missing data, not zero events.

### Prognostic analysis

To identify independent prognostic factors associated with OS and PFS, a Cox proportional hazards regression model was constructed and analyzed. Pathological subtype was collapsed into a binary variable (ALK-positive ALCL vs. non-ALK-positive PTCL) for univariate and multivariate analyses. Univariable analysis indicated that age >60 years, BMI, elevated LDH, and pathological subtype (non-ALK-positive PTCL vs. ALK positive ALCL) significantly influenced OS. Treatment regimens [CHOEP regimen vs. BV plus CHP-like regimen, hazard ratio (HR) 2.260, 95% CI 1.180-4.340; P = 0.012], BMI, elevated LDH, and pathological subtype (non-ALK-positive PTCL vs. ALK positive ALCL) were associated with inferior PFS (**Supplementary Table 2**). However, in the subsequent multivariable analysis (**Supplementary Table 3**), the association between CHOEP therapy and inferior PFS was attenuated and no longer statistically significant (HR 1.865, 95% CI 0.959-3.624; P = 0.066). Ultimately, multivariable analysis demonstrated that BMI (HR 4.069, 95% CI 1.638-10.110, P = 0.003) and elevated LDH (HR 2.997, 95% CI 1.133-7.925, P = 0.027) were independent predictors of poor OS, whereas BMI (HR 2.432, 95% CI 1.232-4.800, P = 0.010), elevated LDH level (HR 2.656, 95% CI 1.349-5.226, P = 0.005), and pathological subtype (non-ALK-positive PTCL vs. ALK positive ALCL, HR 4.322, 95% CI 1.663-11.233, P = 0.003) at diagnosis were independent predictors of inferior PFS.

## Discussion

In this PSM analysis of individual patient data derived from a retrospective multi-center study, we compared the real-world efficacy and safety of the BV plus CHP-like regimen versus the CHOEP regimen among patients with newly diagnosed PTCL. Compared with the CHOEP regimen, the BV plus CHP-like regimen showed no significant differences in ORR, CR, or OS at the end of follow-up. However, the BV plus CHP-like regimen was associated with a longer PFS and a higher DOR rate. Moreover, the BV plus CHP-like regimen was associated with significantly lower hematological toxicity, whereas non-hematological toxicity was comparable between the two groups. To address potential confounding factors such as transplantation, treatment era, BV maintenance, and anthracycline type, several sensitivity analyses were performed. The overall findings remained consistent, with a direction of benefit favoring BV plus CHP-like for PFS and DOR. However, the exclusion of ALK-positive ALCL attenuated the PFS benefit, with the difference in non-ALK-positive PTCL no longer reaching statistical significance (P = 0.110). This suggests that the observed overall PFS advantage may be partly attributable to the favorable prognosis of ALK-positive ALCL. Taken together, this study suggests that the addition of BV to CHP-like chemotherapy may confer superior prognostic benefits compared with CHOEP regimen, without increasing hematological adverse events. Nevertheless, given the potential confounding effect of ALK-positive ALCL, the primary conclusion should be interpreted with caution.

In this study, BV plus CHP-like regimen had a potent ability to achieve responses rapidly and delay disease progression effectively compared with CHOEP regimen. The 2-year PFS rate was significantly higher in the BV plus CHP-like group than that in the CHOEP group (2-year PFS rates: 70.7% vs. 45.3%, P = 0.012). The DOR in the BV plus CHP-like group was significantly prolonged as well (81.9% vs. 63.0%, P = 0.035). Based on the ECHELON-2 trial proving the efficacy of BV in PTCL (16), various retrospective studies have explored the application of BV for treating PTCL in the real world. A retrospective observational single-center study enrolled 19 newly diagnosed PTCL patients treated with BV plus cyclophosphamide, epirubicin and prednisone (BV-CEP), and reported that the BV-CEP regimen had an ORR of 89.5% (CR, 52.7%; PR, 36.8%) (21). Moreover, the BV-CEP regimen was associated with prolonged PFS relative to conventional CHOEP chemotherapy (BV-CEP: not evaluable, CHOEP: 6.5 months; P = 0.026), which is consistent with our findings. Notably, their comparative analysis was limited to a small sample size (16 patients with BV-CEP vs 14 patients with CHOEP) and a short median follow-up (only 6.7 months) without PSM. Another retrospective observational single-center study by Guan et al. enrolled 50 PTCL patients treated with BV-containing regimens (22). Among them, 46 patients were administered cyclophosphamide, epirubicin/liposomal doxorubicin and prednisone, and the remaining 4 patients had additional etoposide. After a median follow-up of 35.2 months, the ORR was 80% (CR, 44%; PR, 36%). The median PFS was 39.7 months (95% CI, 14.7-NA), whereas the median OS was not reached. Given the concurrent use of etoposide, the independent contribution of BV to treatment efficacy could not be fully delineated in their study. Our PSM analysis directly compared BV plus CHP-like with CHOEP, addressing the current gap in comparative efficacy data for these regimens in PTCL. However, our study is limited by the absence of a prospective, randomized head-to-head comparison, which should be addressed in future research.

The BV plus CHP-like regimen was associated with a lower incidence of hematological toxicities than the CHOEP regimen in our study, both for any-grade and for grade ≥ 3 events. The ECHELON-2 trial compared BV plus CHP with CHOP and demonstrated that the safety profile of BV plus CHP remained manageable and very similar to CHOP (16). A meta-analysis proved that BV in combination with other agents was associated with tolerable adverse effects (23). These collective findings resonated well with our study, and validated the safety of BV-based combination therapy. However, CHOEP regimen tends to increase the incidence of AEs. Deng et al. conducted a meta-analysis and found that the CHOEP group had significantly increased AEs (anemia and thrombocytopenia) compared to the CHOP group (24). A retrospective, observational and single-center study involving 124 nodal PTCL patients reported that the addition of etoposide to the CHOP backbone showed no survival benefit and was associated with high toxicity (25). Collectively, CHOEP carries higher hematological toxic risks with poor tolerability while BV plus CHP-like did not aggravate hematologic adverse events and retained a favorable safety profile in patients with PTCL.

The results in our study showed favorable OS outcomes compared with other PTCL-related studies, and the potential contributing factors were analyzed as follows (16, 25). First, the patients enrolled in our study were predominantly under 60 years of age, accounting for 70% of the study population. As an established independent prognostic factor for PTCL, younger age confers better physical reserve, treatment tolerance, and fewer comorbidities, collectively improving survival (19). Second, 13.3% of patients received HSCT, which substantially improved survival. Third, the relatively high proportion of ALK-positive ALCL in both arms may have contributed to the favorable OS in our study, since ALK-positive ALCL is associated with a favorable prognosis (11, 16). Given the small sample size in our study, large-scale prospective controlled studies are warranted for further validation in future investigations.

Of note, PFS and DOR favored the BV plus CHP-like regimen, whereas OS and final response did not differ significantly between arms. Several factors may account for this discrepancy. First, the disease mix was unbalanced between arms, with a relatively high proportion of ALK-positive ALCL in both groups. Second, the sample size was limited, and the number of OS events was relatively small (19 events in total), which restricted the statistical power to detect an OS difference. Third, post-progression therapies, including HSCT and novel agents, were permitted and may have attenuated the impact of the PFS difference on OS. Taken together, these factors suggest that the observed PFS/DOR benefit should be interpreted as hypothesis-generating rather than as evidence of a definitive OS benefit, and longer follow-up is warranted to confirm these findings.

Several limitations should be addressed. First, this was a retrospective study with a relatively small sample size, limiting the generalizability of the results. Second, despite performing PSM, residual imbalances, like extranodal involvement, LDH level, and pathological subtypes, may introduce potential confounding. Of note, conducting stratified analyses for these variables would substantially decrease sample size and event counts within subgroups, thus compromising statistical power in this small cohort. Third, the median follow-up of the overall cohort was 27.2 months, and follow-up time was uneven between treatment arms. Accordingly, the apparent long-term plateaus observed in survival curves should be interpreted with caution. Fourth, CD30 data were not uniformly available and were not included in the propensity score model, so CD30-related confounding remains an important residual limitation. The CD30-positive subgroup was also unbalanced between groups (41/45 vs. 15/45). These results should therefore be considered exploratory.

## Conclusions

In conclusion, our multi-center real-world data and PSM analysis demonstrated that the BV plus CHP-like regimen conferred superior PFS and DOR relative to the CHOEP regimen, suggesting a potent ability to effectively delay disease progression. However, this PFS benefit was attenuated in non-ALK-positive PTCL patients and should be interpreted with caution. Furthermore, this analysis revealed a lower incidence of hematological toxicities with the BV plus CHP-like regimen compared with the CHOEP regimen. Collectively, these findings support the BV plus CHP-like regimen as a promising first-line therapeutic option for PTCL, which warrants further investigation.

## Data Availability

The raw data supporting the conclusions of this article will be made available by the authors, without undue reservation.
