# Supplementary material for: Brentuximab vedotin plus CHP-like versus CHOEP regimen for the first-line treatment of PTCL patients: a multi-center propensity-matched retrospective study
Source: Front Immunol. 2026 Oct 21;17:1973646. doi: 10.3389/fimmu.2026.1973646 (PMC13731321; doi:10.3389/fimmu.2026.1973646)
Supplement: Supplementary file 1 [file DataSheet1.pdf]

Patient enrollment

Inclusion criteria:

(1) Patients with newly diagnosed PTCL patients between January 2014 and December 2022 from the First Affiliated Hospital of Zhejiang University School of Medicine (The follow-up deadline was June 30, 2023.) and between January 2020 and December 2024 from Wuhan Union Hospital, Huazhong University of Science and Technology (The follow-up deadline was October 31, 2025) were allocated.

(2) Relative clinical data before treatment were available for statistical analysis.

(3) Patients underwent bone marrow test to evaluate the status of bone marrow and were available to evaluate the IPI and PIT.

Exclusion criteria:

(1) Patients with composite lymphoma

(2) Patients who had other uncured malignancy cancers or severe infection before enrollment.

(3) Those with data missing.

Matching method

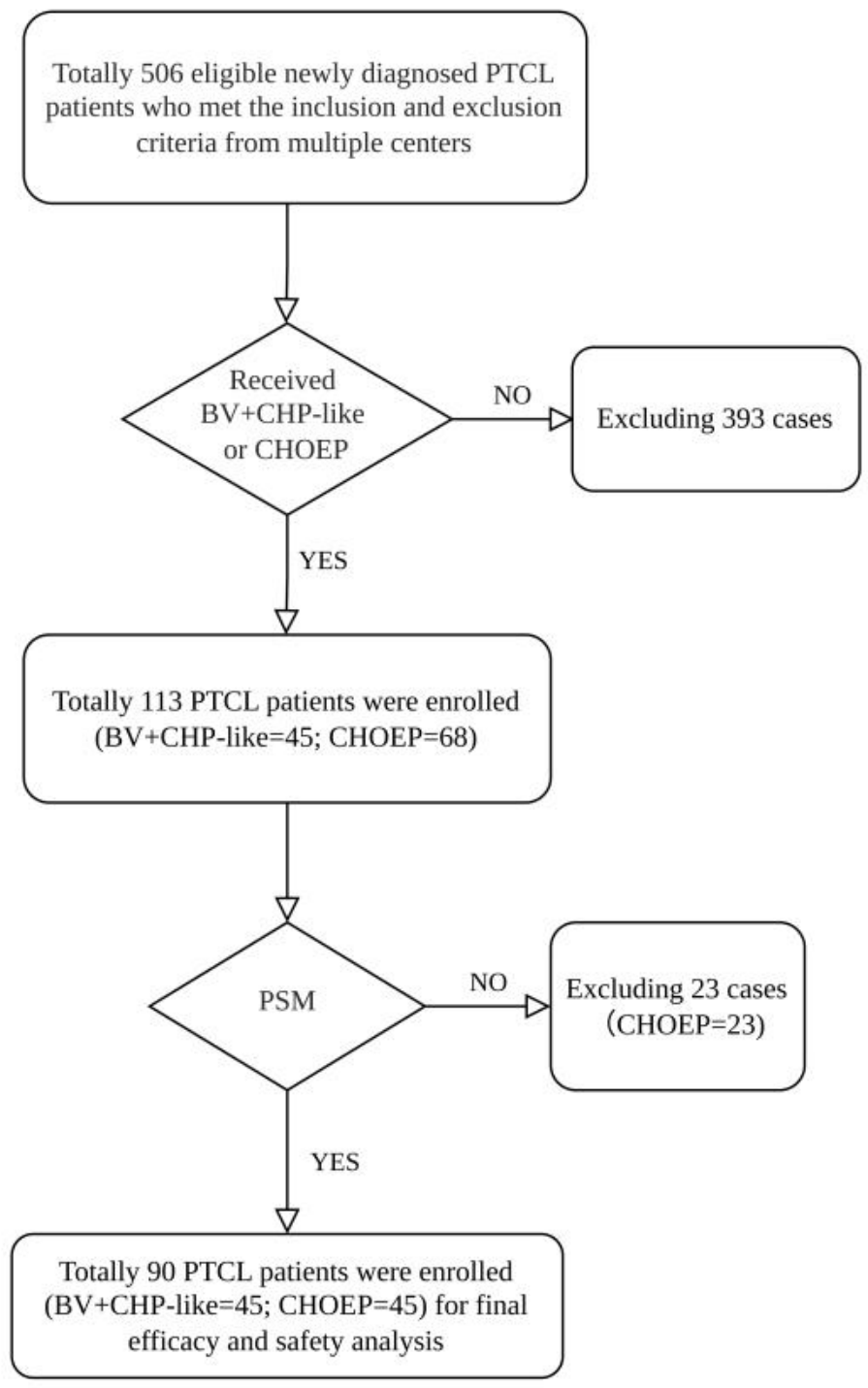

Figure S1. Flowchart

Propensity score matching (PSM) was performed using a 1:1 optimal matching approach, with matching variables including gender, age, disease stage, bone marrow infiltration (BMI), extra-nodal involvement, Eastern Cooperative Oncology Group performance status (ECOG PS) and PTCL subtypes. After matching, two balanced cohorts of 45 patients each were generated for the BV plus CHP-like and CHOEP groups, respectively.

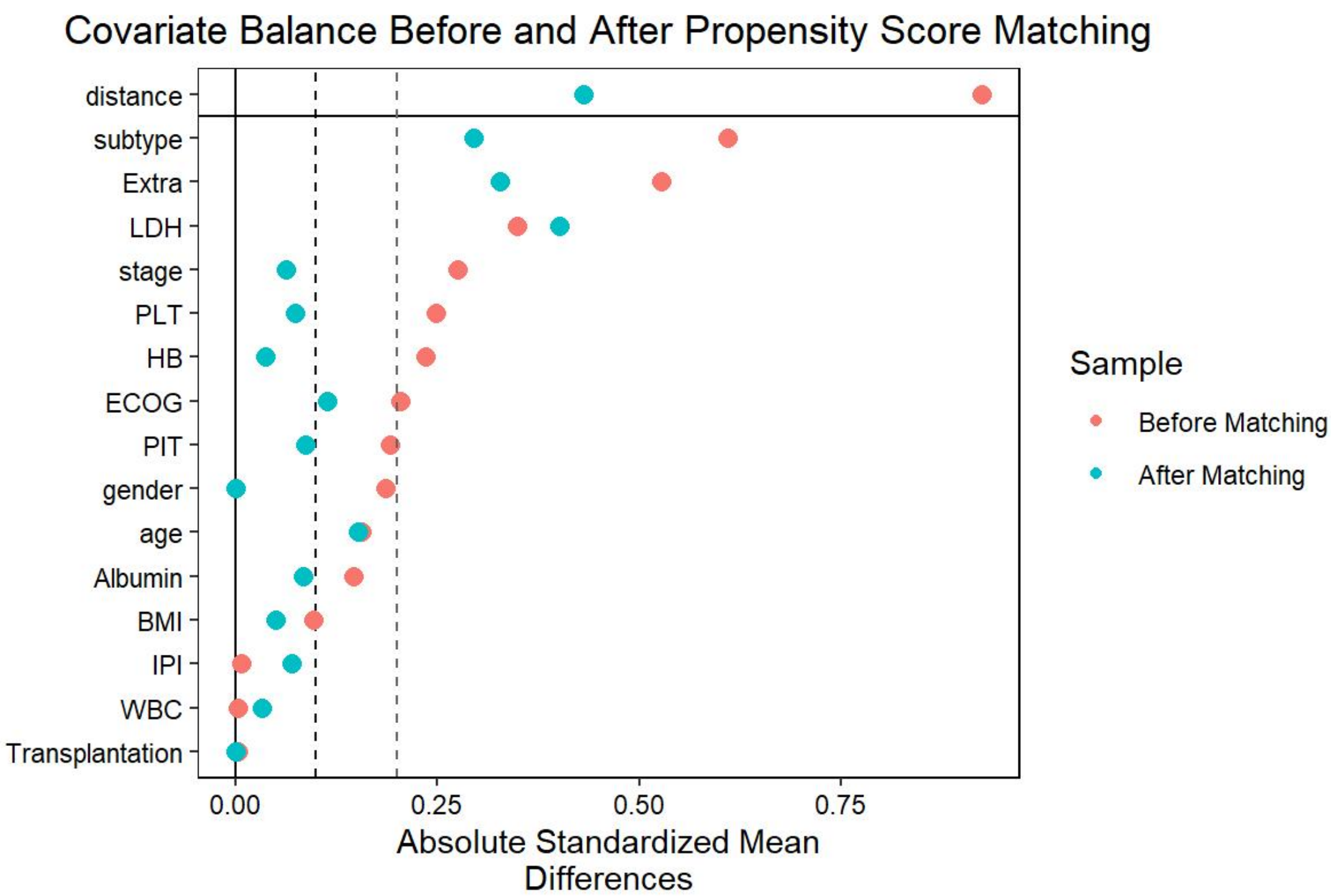

Figure S2 Covariate balance before and after propensity score matching.

The Love plot displays the standardized mean differences (SMDs) for each baseline covariate before (red dots) and after (blue dots) propensity score matching. Subtype was included in the propensity score model as a continuous term to preserve statistical power given the limited sample size. IPI and PIT (as a continuous term in Love plot) were not included in the propensity score model and were presented in theTable S1 for descriptive purposes only. The vertical dashed lines represent the acceptable threshold of 0.1 and 0.2.

Abbreviations: Extra, extranodal sites; LDH, lactate dehydrogenase; PLT, platelet count; HB, hemoglobin; ECOG, Eastern Cooperative Oncology Group performance status; PIT, prognostic index for PTCL;BMI, bone marrow infiltration; IPI, International Prognostic Index; WBC, white blood cell.

Number of events:

In the matched cohort (n = 90), there were 40 PFS events (14 in the BV plus CHP-like group and 26 in the CHOEP group) and 19 OS events (8 in the BV plus CHP-like group and 11 in the CHOEP group). Among patients who achieved a response, 18 DOR events occurred (6 in the BV plus CHP-like group and 12 in the CHOEP group).

Anthracycline type：

In the matched cohort, the distribution of anthracycline type was as follows: Doxorubicin: 40 patients (88.9%) in the BV plus CHP-like; Liposomal doxorubicin: 3 patients (6.7%) in the BV plus CHP-like group; Epirubicin: 2 patients (4.4%) in theBV plus CHP-like group. The decision of anthracycline type was made by the treating physician based on clinical judgment, and patient preference; it was not protocol-mandated.

Definitions of PFS, OS, and DOR

PFS was defined as the time from treatment initiation to documented disease progression or death from any cause, whichever occurred first. Patients who were alive without progression at the last adequate tumor assessment were censored on the date of the last adequate assessment. OS was defined as the time from treatment initiation to death from any cause. Patients who were still alive at the last follow-up were censored on the last known alive date. DOR was analyzed only in responders (patients achieving CR or PR). DOR was defined as the time from first documented response to documented progression or death. Responders who were alive without progression were censored on the date of the last adequate tumor assessment. Patients lost to follow-up were censored at the last date on which they were known to be alive and progression-free (for PFS/DOR) or the last known alive date (for OS).

Timing and definition of "interim" and "end-of-follow-up" response

Interim response was assessed after 3 or 4 cycles, using PET-CT according to the Deauville 5-point scale (5-PS) or CT according to RECIST v1.1. Response assessment was based on the Lugano criteria (2014). End-of-follow-up evaluation, including survival outcome and disease status, was performed at the study cut-off date. Patients whose interim assessment suggested a response but who later progressed were classified according to their final assessment rather than the interim result.

CD 30 expression

CD30 status	Total	BV plus CHP-like group (n=45)	CHOEP group (n= 45)
**Positive CD30 (any expression)**	56	41	15
Reported as positive only	31	20	11
Reported as partly positive	18	14	4
Reported with specific percentage	7	7	0
10% positive	1	1	0
20% positive	2	2	0
30% positive	1	1	0
40% positive	1	1	0
50% positive	1	1	0
60% positive	1	1	0
**Negative CD30**	24	3	21
**Missing data**	10	1	9

Note: CD30 positivity was defined as any documented CD30 expression. In most cases, CD30 status was reported qualitatively (positive, partly positive, or negative) without a specific percentage; quantitative CD30 expression data were therefore not available for all patients.

Patients were defined as CD30-positive if any CD30 positivity was reported. Among

CD30-negative PTCL patients, no significant differences in OS (P = 0.18) or PFS (P = 0.69) were observed between the BV plus CHP-like group and the CHOEP group. Similarly, among CD30-positive PTCL patients, no significant differences in OS (P = 0.65) or PFS (P = 0.48) were found between the two treatment groups. These subgroup analyses should be interpreted with caution given the limited sample size and the substantial imbalance in CD30 distribution between treatment groups (CD30-positive: 41/45 in the BV plus CHP-like group vs. 15/45 in the CHOEP group).

Survival outcomes in the time-matched cohort

To eliminate the confounding effect of treatment era, we excluded patients who received CHOEP before 2020, resulting in a final cohort of 45 patients in the BV plus CHP-like group and 35 patients in the CHOEP group. The median follow-up duration was 25.8 months (95% CI: 19.4−32.6). At the 2-year mark, the OS rate was 81.0% (95% CI: 68.8%–95.3%) in the BV plus CHP-like group compared with 72.9% (95% CI: 58.0%–91.7%) in the CHOEP group, with no statistically significant difference observed (P = 0.410, Figure S3A). In contrast, the BV plus CHP-like group demonstrated a significantly superior PFS. The 2-year PFS rate was 70.7% (95% CI: 58.5%–85.5%) for the BV plus CHP-like group versus 44.0% (95% CI: 30.0%–64.4%) for the CHOEP group (P = 0.007, Figure S3B). Furthermore, the DOR was also significantly prolonged in the BV plus CHP-like group (P = 0.020, Figure S3C). Overall, these findings were consistent with the primary analysis, with significantly better PFS and DOR in the BV plus CHP-like group.

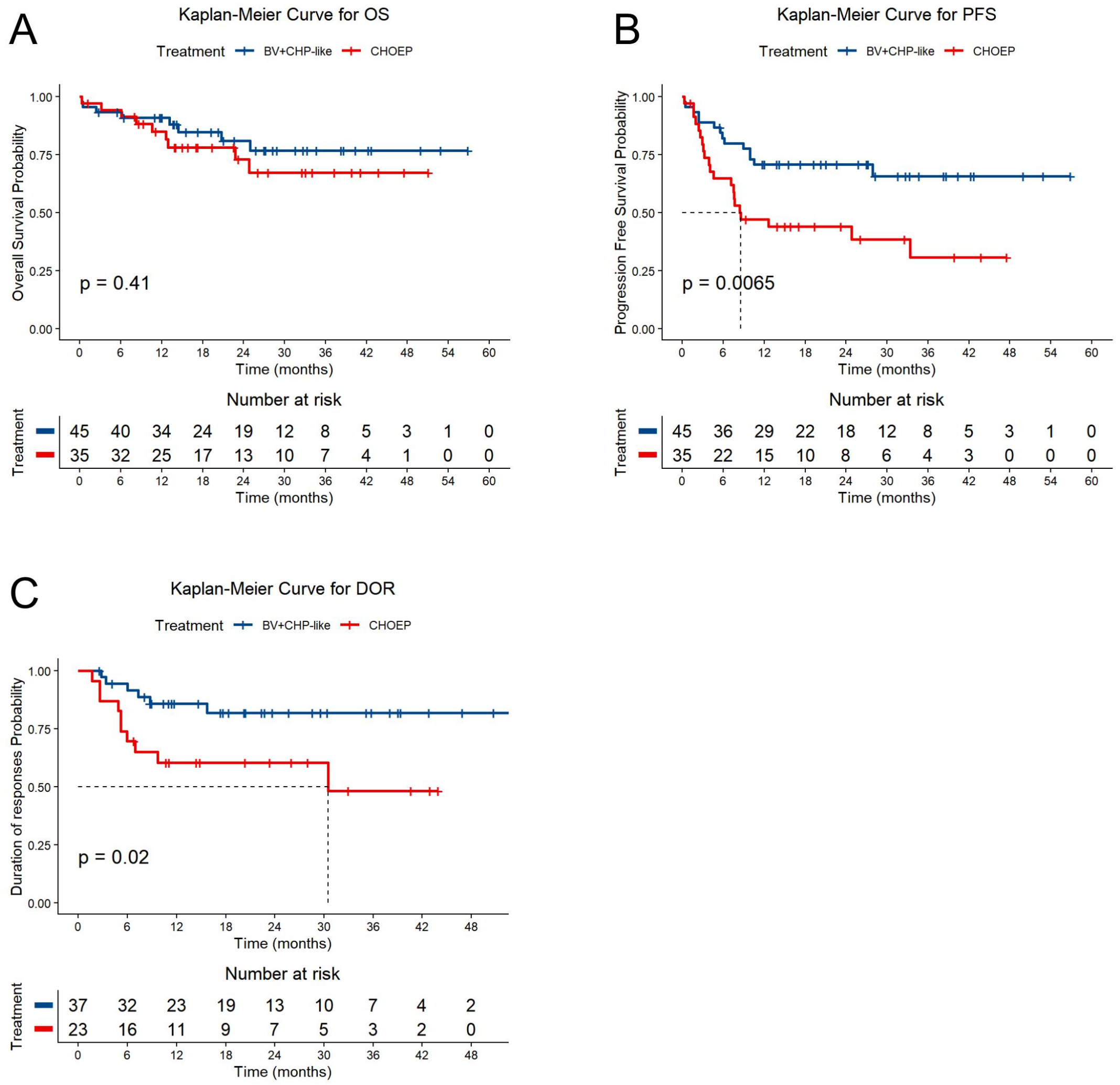

Figure S3. Survival outcomes in the matched cohort excluding patients treated before 2020. (A) Kaplan-Meier curves for overall survival (OS). (B) Kaplan-Meier curves for progression-free survival (PFS). (C) Kaplan-Meier curves for duration of response (DOR) among responders. P-values were calculated using the log-rank test.

Survival outcomes in patients without BV maintenance

To further eliminate the potential confounding effect of BV maintenance therapy, we performed a sensitivity analysis by excluding 10 patients who received BV maintenance. In this subset, the OS remained comparable between the two groups (P = 0.350, Figure S4A). At the 24-month landmark, the PFS rate was 76.9% (95% CI, 64.0%−92.3%) in the BV plus CHP-like group versus 45.3% (95% CI, 32.7%−62.7%) in the CHOEP group. Notably, the PFS benefit significantly favored the BV plus CHP-like group compared to the CHOEP group (P = 0.009, Figure S4B). Similarly, DOR was significantly longer in the BV plus CHP-like group (P = 0.016, Figure S4C). Overall, these findings were consistent with the primary analysis, demonstrating significantly improved PFS and DOR in the BV plus CHP-like group.

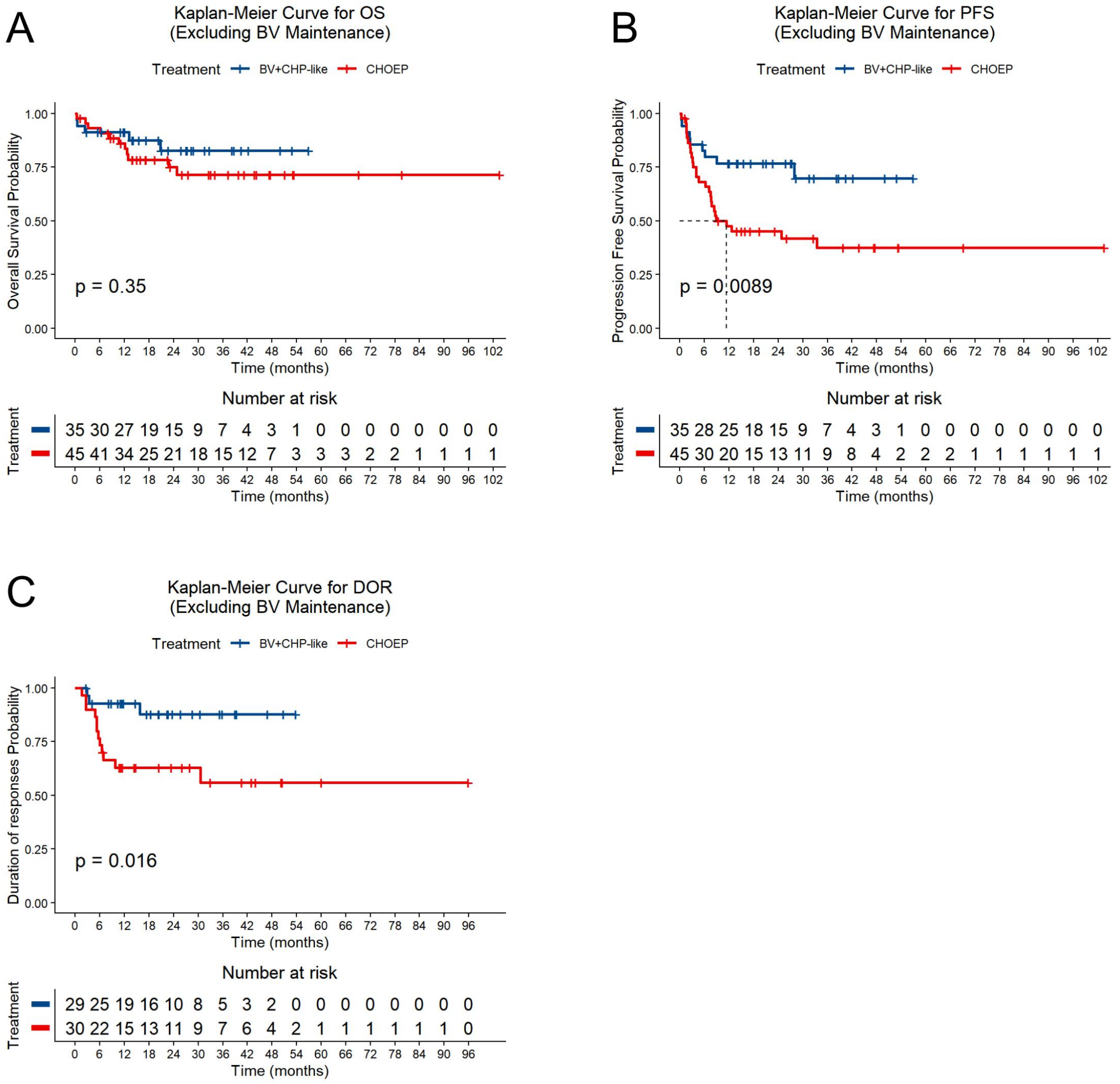

Figure S4. Survival outcomes in patients without BV maintenance.
(A) Kaplan-Meier curves for overall survival (OS). (B) Kaplan-Meier curves for progression-free survival (PFS). (C) Kaplan-Meier curves for duration of response (DOR) among responders. P-values were calculated using the log-rank test.

Subgroup survival analyses in patients without transplantation

To eliminate the confounding effect of transplantation, patients who underwent transplantation (n = 12) were not included in this analysis, leaving 39 patients in each group. For OS, the 24-month survival rate was 84.9% (95% CI 73.0%–98.7%) in the BV plus CHP-like group versus 71.0% (95% CI 57.1%–88.3%) in the CHOEP group, although the difference was not statistically significant (P = 0.150, Figure S5A). For PFS, the 24-month PFS rate was significantly higher in the BV plus CHP-like group than in the CHOEP group (74.0% vs. 36.5%; P = 0.002, Figure S5B). Similarly, the 24-month DOR rate was 88.4% (95% CI 76.8%–100.0%) in the BV plus CHP-like group versus 53.6% (95% CI 36.8%–78.1%) in the CHOEP group (P = 0.002, Figure S5C). These findings were consistent with the primary analysis, confirming the PFS and DOR benefits of the BV plus CHP-like regimen in patients who did not undergo transplantation.

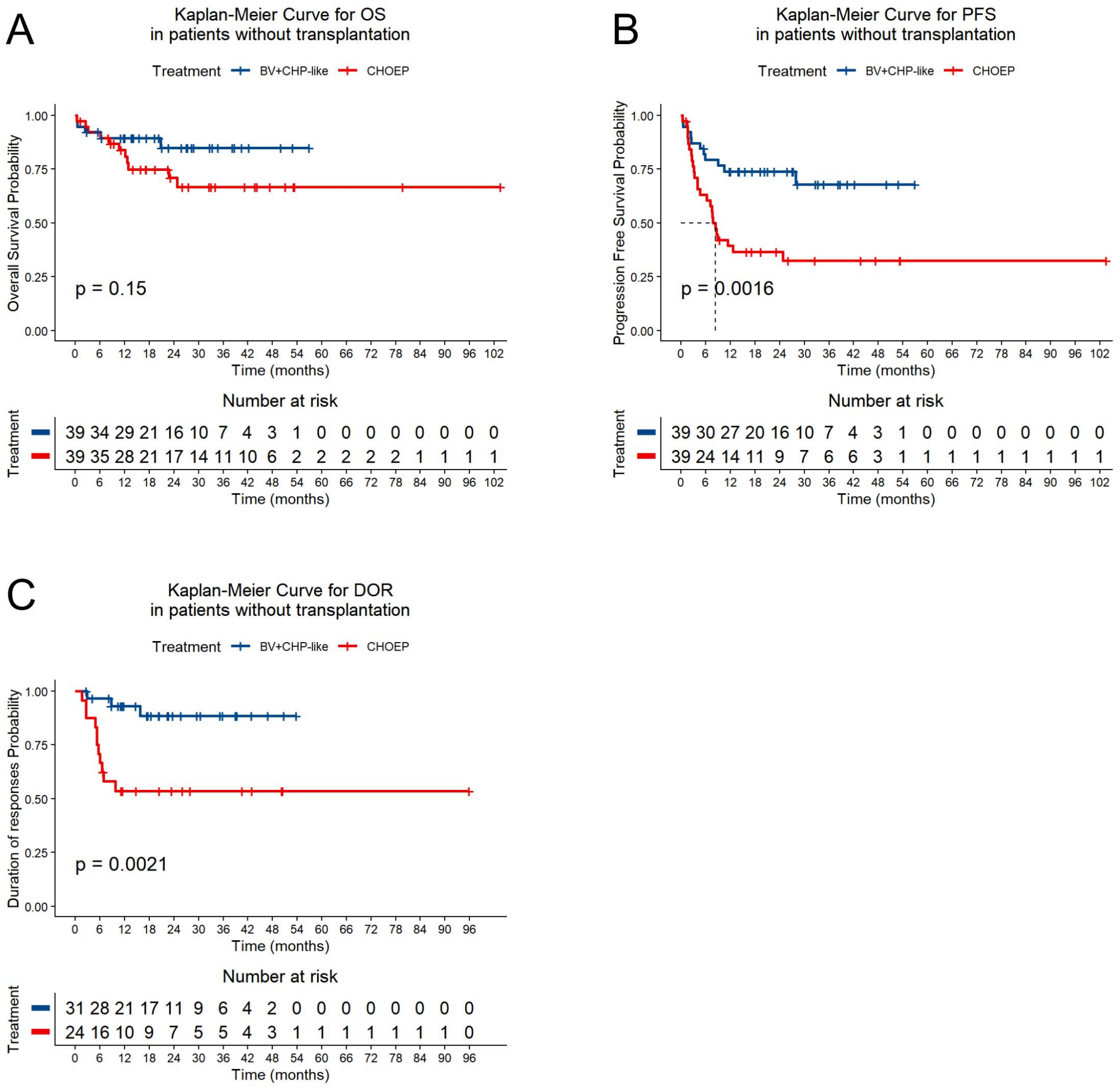

Figure S5. Survival outcomes in patients without transplantation.
(A) Kaplan-Meier curves for overall survival (OS). (B) Kaplan-Meier curves for progression-free survival (PFS). (C) Kaplan-Meier curves for duration of response (DOR) among responders. P-values were calculated using the log-rank test.

Subgroup survival analyses in patients receiving conventional BV plus CHP versus CHOEP

To eliminate the confounding effect of anthracycline type, we excluded patients who received liposomal doxorubicin (n = 3) or epirubicin (n = 2), resulting in a final cohort of 40 patients in the BV plus CHP group and 45 patients in the CHOEP group. Kaplan-Meier curves for OS, PFS, and DOR are shown in Figure S6. At 24 months, OS was 79.1% (95% CI 66.1%–94.7%) in the BV plus CHP-like group versus 75.1% (95% CI 62.7%–90.1%) in the CHOEP group (Figure S6A, P = 0.710). PFS was significantly higher in the BV plus CHP-like group (72.1%, 95% CI 59.3%–87.6%) than in the CHOEP group (45.3%, 95% CI 32.7%–62.7%; Figure S6B, P = 0.007). DOR was also significantly better with BV plus CHP-like than with CHOEP (Figure S6C; P = 0.030). Overall, the BV plus CHP-like group showed a consistent advantage over the CHOEP group, with significantly better PFS and DOR and a numerically

higher but non-significant OS, consistent with the primary analysis.

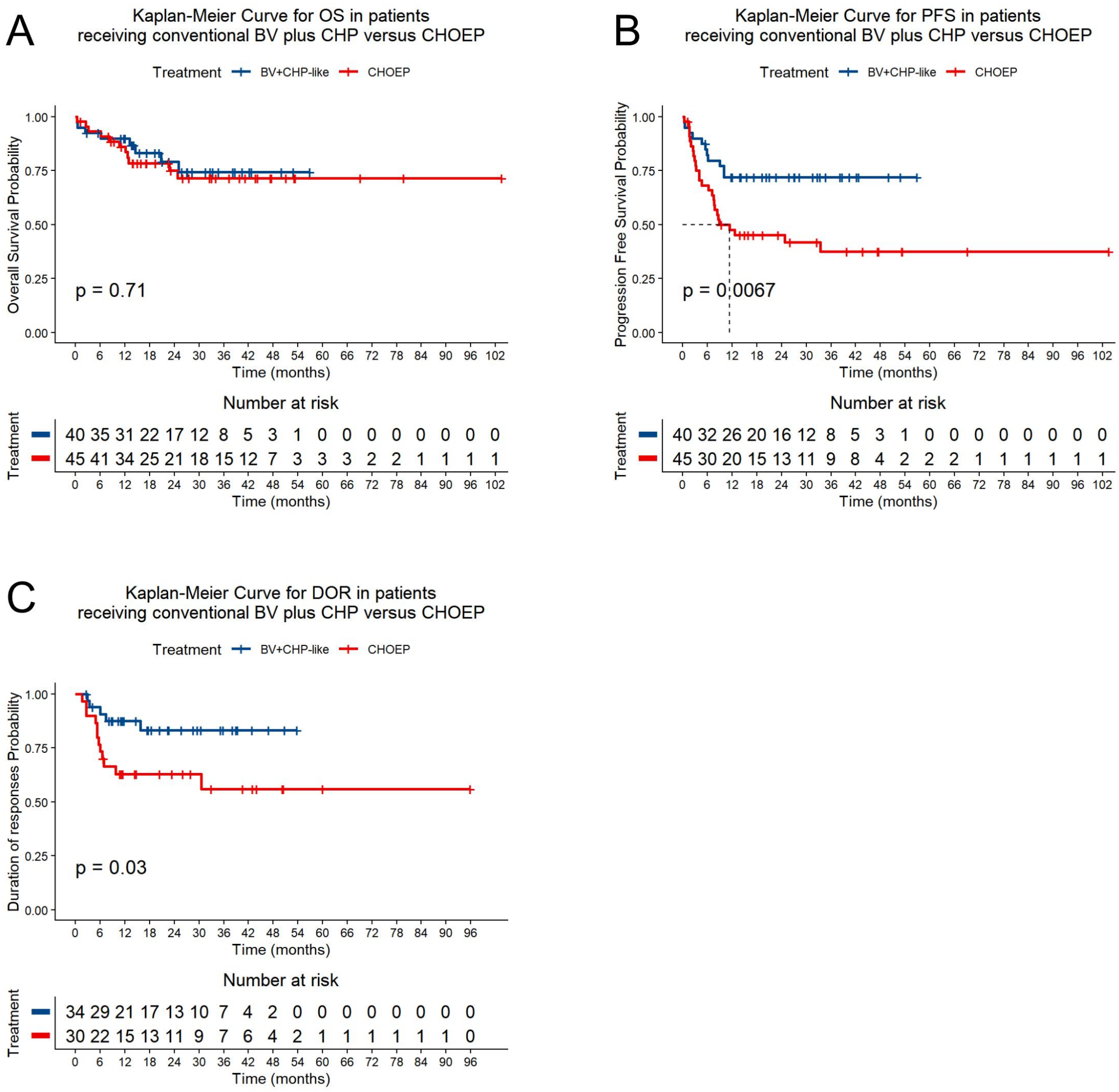

Figure S6. Survival outcomes in patients receiving conventional BV plus CHP versus CHOEP.

(A) Kaplan-Meier curves for overall survival (OS). (B) Kaplan-Meier curves for progression-free survival (PFS). (C) Kaplan-Meier curves for duration of response (DOR) among responders. P-values were calculated using the log-rank test.

Subgroup survival analyses by center

To evaluate the potential impact of center-specific factors, we performed a stratified survival analysis. In patients from Wuhan Union Hospital, the OS remained comparable between the two groups (P = 0.120, Figure S7A). However, the BV plus CHP-like group demonstrated a significantly superior PFS, with a 2-year PFS rate of 78.9% (95% CI: 65.2%–95.4%) compared to 36.4% (95% CI: 16.6%–79.5%) in the CHOEP group (P = 0.014, Figure S7B). In patients from Zhejiang University First Hospital, the 2-year OS rate was 81.2% (95% CI, 68.7%–96.0%) versus 68.6% (95% CI, 46.6%–100%) (P = 0.12, Figure S7C), and the 2-year PFS rate was 56.2% (95% CI, 36.5–86.7%) versus 48.3% (95% CI, 33.9%–68.8%, P = 0.620, Figure S7D). Overall, the PFS benefit of the BV plus CHP-like regimen was consistently observed

across both centers, although the difference did not reach statistical significance in the Zhejiang cohort, likely due to the limited sample size and relatively low statistical power in this subgroup, as well as potential differences in patient characteristics or subsequent therapies between centers.

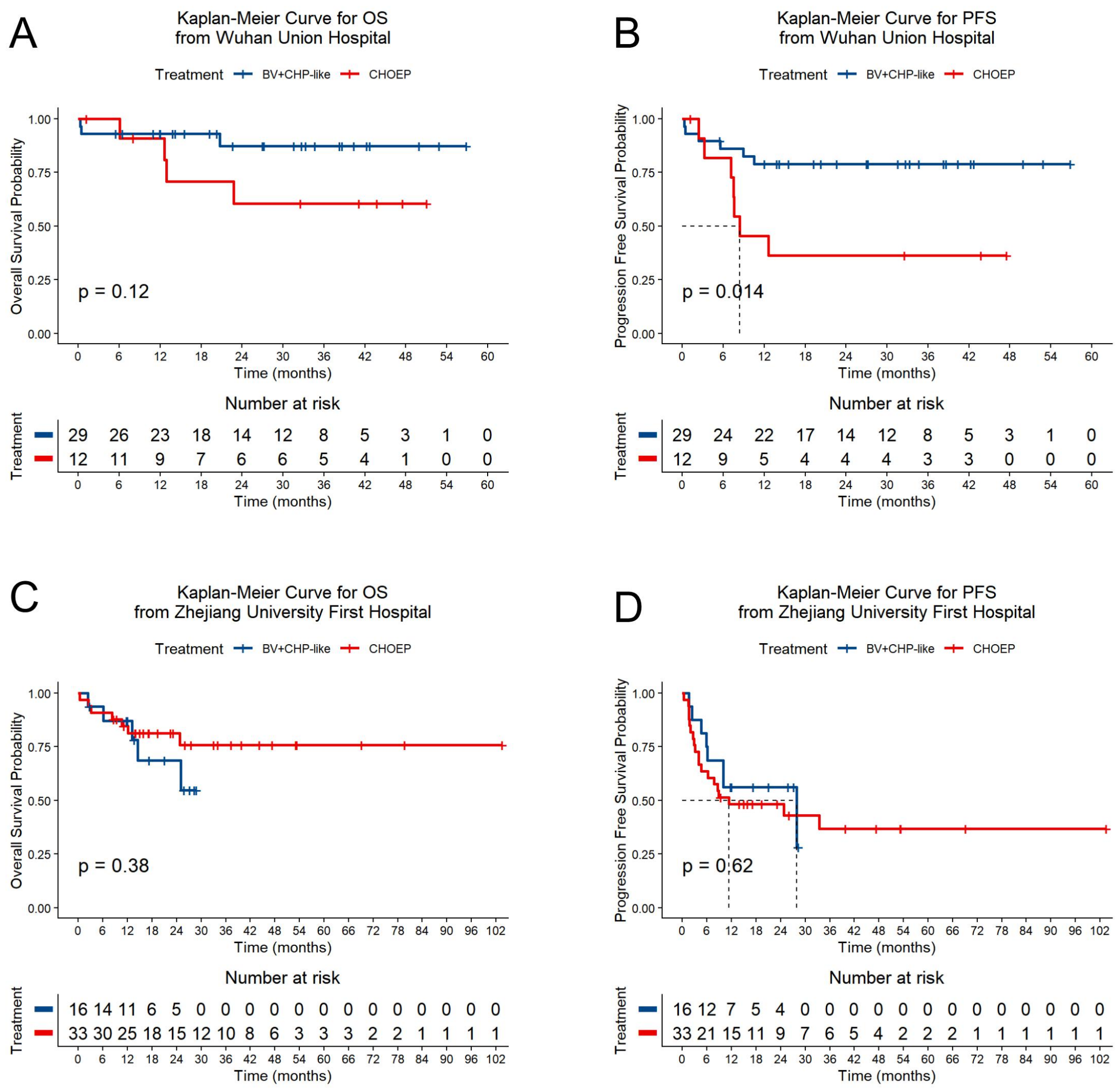

Figure S7. Survival outcomes stratified by treatment center.
(A, B) Kaplan-Meier curves for overall survival (OS) and progression-free survival (PFS) in patients from Wuhan Union Hospital. (C, D) Kaplan-Meier curves for OS and PFS in patients from Zhejiang University First Hospital. P-values were calculated using the log-rank test.

Subgroup survival analyses by LDH level

Subgroup analyses were further stratified by baseline LDH level. Kaplan-Meier curves for OS and PFS in patients with higher-level LDH are shown in Figure S8 (A, B), and those for lower-level LDH in Figure S8 (C, D). In the higher-level LDH subgroup, no significant differences were observed between the BV plus CHP-like and CHOEP groups for OS (P = 0.430) or PFS (P = 0.530). At 24 months, OS rates were 61.1% versus 73.7%, and PFS rates were 41.3% versus 32.5%, respectively

(CHOEP vs. BV plus CHP-like). In the lower-level LDH subgroup, OS did not differ significantly between groups (P = 0.220), whereas PFS showed a non-significant trend favoring BV plus CHP-like (P = 0.055). At 24 months, OS rates were 91.2% versus 77.6%, and PFS rates were 86.2% versus 60.0%, respectively. Overall, no statistically significant differences were observed in either LDH stratum, although a numerical trend toward improved outcomes with BV plus CHP-like therapy was observed in the lower-LDH subgroup. Given the limited sample size, larger prospective studies are warranted to confirm these findings.

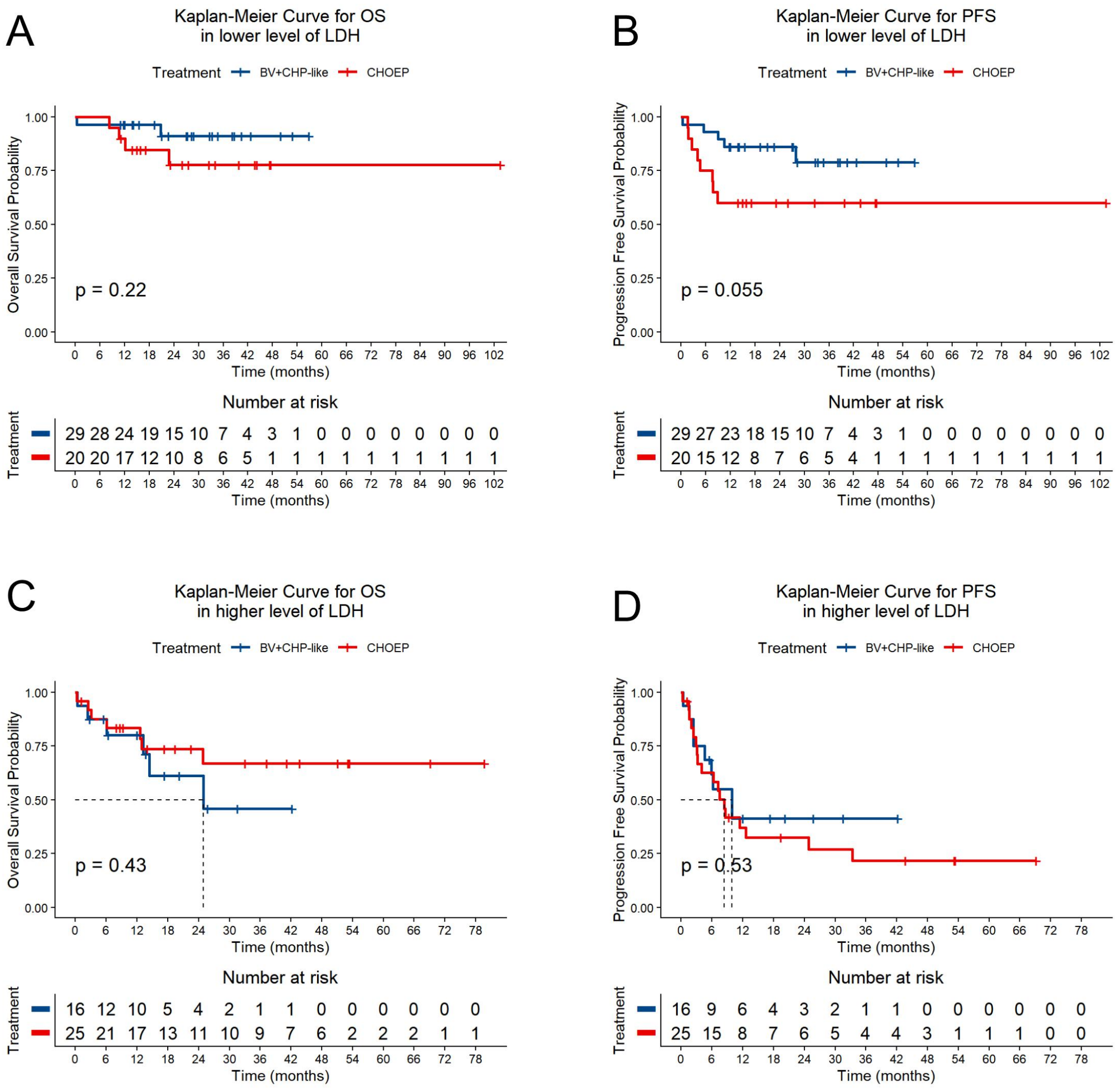

Figure S8. Survival outcomes stratified by LDH level.
(A, B) Kaplan-Meier curves for overall survival (OS) and progression-free survival (PFS) in patients with higher level of LDH. (C, D) Kaplan-Meier curves for OS and PFS in patients with lower level of LDH. P-values were calculated using the log-rank test.

Subgroup survival analyses by the number of extranodal involvement

Subgroup analyses were further stratified by baseline extranodal involvement. Kaplan-Meier curves for OS and PFS in patients with extranodal involvement >1 are shown in Figure S9 (A, B), and those with extranodal involvement ≤ 1 in Figure S9

(C, D). In the patients with extranodal involvement >1, no significant differences were observed between the BV plus CHP-like and CHOEP groups for OS (P = 0.720) or PFS (P = 0.900). In the patients with extranodal involvement ≤ 1, OS did not differ significantly between groups (P = 0.600), whereas PFS showed a significant different favoring BV plus CHP-like (P = 0.003). At 24 months, OS rates were 83.8% versus 77.7%, and PFS rates were 80.2% versus 45.6%, respectively (BV plus CHP-like vs CHOEP). These findings suggest that the PFS benefit of BV plus CHP-like over CHOEP was more pronounced in patients with extranodal involvement ≤ 1, whereas no significant difference was observed in those with extranodal involvement > 1. Given the small sample sizes, these findings should be interpreted with caution and warrant further validation in larger prospective cohorts.

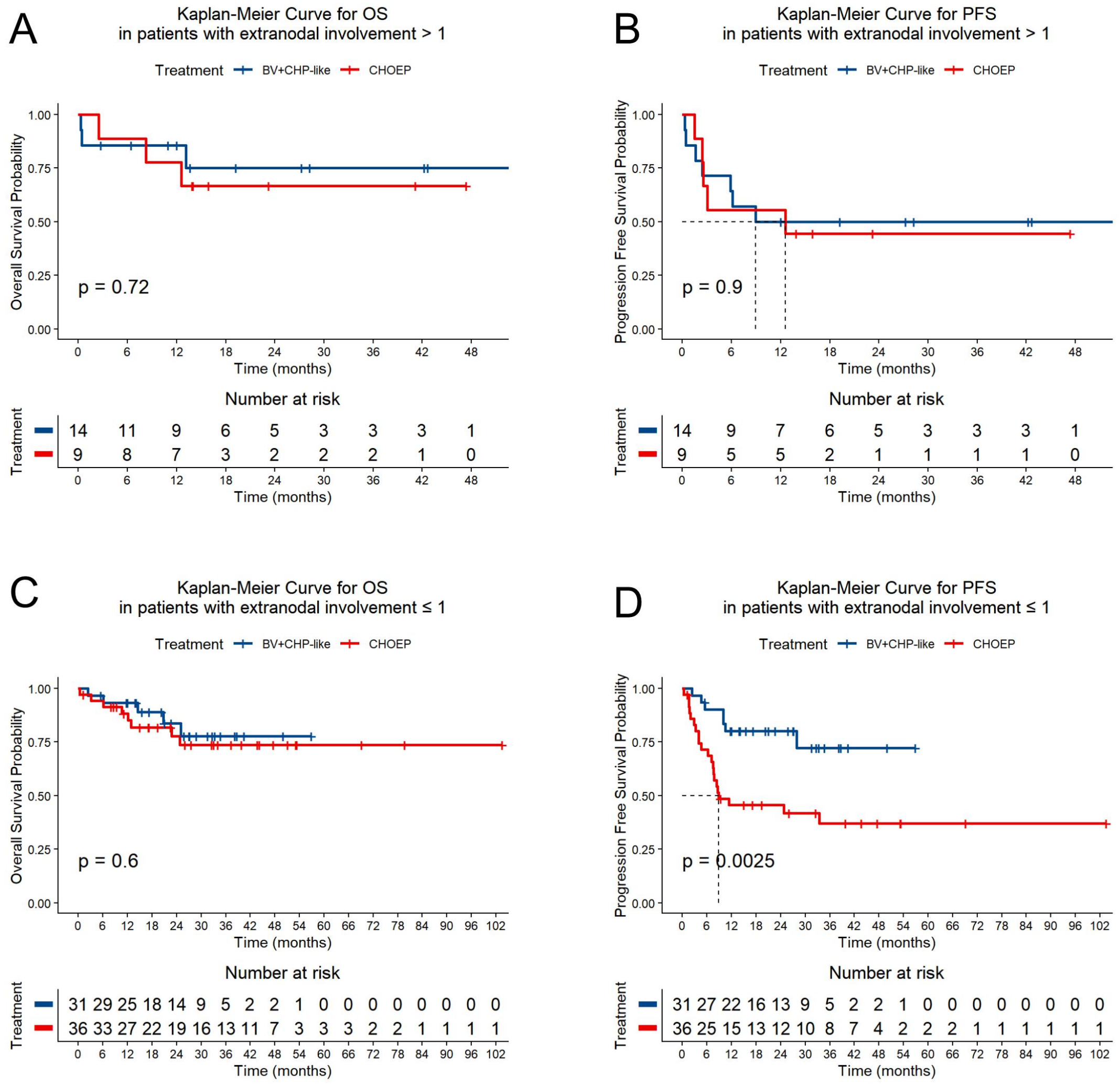

Figure S9. Survival outcomes stratified by the number of extranodal involvement.
(A, B) Kaplan-Meier curves for overall survival (OS) and progression-free survival (PFS) in patients with extranodal involvement > 1. (C, D) Kaplan-Meier curves for OS and PFS in patients with extranodal involvement ≤ 1. P-values were calculated using the log-rank test.

Table S1
Covariate balance of treated (BV plus CHP-like) and control (CHOEP) groups before and after matching

Variable	Category	Treated Mean (BV plus CHP-like)	Control Mean (CHOEP)	SMDs
Distance	Before Matching	0.5082	0.6637	0.9238
	After Matching	0.5082	0.5807	**0.4305**
Male	Before Matching	0.6222	0.5294	-0.1859
	After Matching	0.6222	0.6222	0.0000
Subtype*	Before Matching	3.0000	2.2206	-0.6091
	After Matching	3.0000	2.6222	**-0.2952**
Age	Before Matching	0.3333	0.2647	-0.1556
	After Matching	0.3333	0.2667	**-0.1511**
Stage	Before Matching	0.7556	0.8529	0.2750
	After Matching	0.7556	0.7778	0.0627
BMI	Before Matching	0.2222	0.2647	0.0963
	After Matching	0.2222	0.2444	0.0504
Extra	Before Matching	0.3111	0.1324	-0.5275
	After Matching	0.3111	0.2000	**-0.3279**
ECOG PS	Before Matching	0.1111	0.1912	0.2036
	After Matching	0.1111	0.0667	**-0.1130**
LDH	Before Matching	0.3556	0.5294	0.3483
	After Matching	0.3556	0.5556	**0.4007**
Albumin	Before Matching	38.6156	37.5172	-0.1459
	After Matching	38.6156	37.9860	-0.0836

Variable	Category	Treated Mean (BV plus CHP-like)	Control Mean (CHOEP)	SMDs
WBC	Before Matching	9.1431	9.1043	-0.0037
	After Matching	9.1431	9.4856	0.0324
HB	Before Matching	123.1333	117.0735	-0.2358
	After Matching	123.1333	122.2000	-0.0363
PLT	Before Matching	223.3111	199.4559	-0.2482
	After Matching	223.3111	216.2000	-0.0740
IPI*	Before Matching	2.0222	2.0294	0.0075
	After Matching	2.0222	1.9556	-0.0693
PIT*	Before Matching	1.0222	1.2206	0.1918
	After Matching	1.0222	1.1111	0.0859
AITL	Before Matching	0.2222	0.4706	0.4976
	After Matching	0.2222	0.3333	**0.2226**
PTCL-TFH	Before Matching	0.0667	0.0882	0.0760
	After Matching	0.0667	0.0444	-0.0783
PTCL NOS	Before Matching	0.2000	0.1912	-0.0224
	After Matching	0.2000	0.2889	**0.2260**
ALK positive ALCL	Before Matching	0.4000	0.1912	-0.5310
	After Matching	0.4000	0.2889	**-0.2826**
ALK negative ALCL	Before Matching	0.1111	0.0588	-0.2222
	After Matching	0.1111	0.0444	**-0.2833**
Transplantation	Before Matching	0.1333	0.1324	-0.0029
	After Matching	0.1333	0.1333	0.0000

Note: *Subtype, IPI and PIT were treated as continuous scores for SMD calculation; For continuous variables (Albumin, WBC, HB, PLT), SMDs were calculated based on mean values to assess balance, whereas Table 1 reports medians (range) due to non-normal distributions.

subtype-specific proportions and SMDs before and after matching (AITL, PTCL-TFH, PTCL NOS, ALK positive ALCL and ALK negative ALCL) are provided.

Abbreviations: SMDs: standardized mean differences; Extra, extranodal sites; BMI, bone marrow infiltration; ECOG PS, Eastern Cooperative Oncology Group performance status; LDH, lactate dehydrogenase; WBC, white blood cell; HB, hemoglobin; PLT, platelet count; IPI, International Prognostic Index; PIT, prognostic index for PTCL; AITL, angioimmunoblastic T-cell lymphoma; PTCL-TFH, nodal T-follicular helper (TFH) cell lymphoma; PTCL NOS, peripheral T-cell lymphoma, not otherwise specified; ALCL, anaplastic large cell lymphoma

Table S2
Univariable analysis for the overall survival and progression-free survival.

Prognostic factors	OS		PFS	
	**HR (95%CI)**	**Cox P-value**	**HR (95%CI)**	**Cox P-value**
Male	0.848 (0.341-2.110)	0.723	1.790 (0.895-3.590)	0.099
Age >60 years	2.770 (1.120-6.840)	**0.028**	1.660 (0.873-3.150)	0.122
Ann Arbor stage III/IV	3.100 (0.715-13.400)	0.131	2.140 (0.897-5.120)	0.086
ENSs ≥2	1.620 (0.613-4.270)	0.331	1.500 (0.763-2.950)	0.239
BMI	4.400 (1.770-10.900)	**0.001**	2.570 (1.320-5.000)	**0.005**
ECOG PS ≥2	2.600 (0.755-8.930)	0.130	1.970 (0.770-5.030)	0.157
Elevated LDH	3.260 (1.240-8.590)	**0.017**	3.610 (1.860-7.030)	**<0.001**
Subtype				
ALK positive ALCL	Reference		Reference	
Non-ALK-positive PTCL	Not estimable*	**< 0.001** †	5.720 (2.230-14.700)	**<0.001**
Treatment				
BV plus CHP-like	Reference		Reference	
CHOEP	1.320 (0.532-3.290)	0.547	2.260 (1.180-4.340)	**0.012**

Note: *HR not estimable due to complete separation (no OS events in the ALK positive ALCL subgroup). †Log-rank P value.

HR >1 indicates an increased risk of death (OS) or progression/death (PFS) for the category listed compared with the reference category. The reference categories for each variable are: Female (for Male), Age ≤ 60 (for Age > 60), Ann Arbor stage I/II (for stage III/IV), ENSs < 2 (for ENSs ≥ 2), No BMI (for BMI), ECOG PS < 2 (for ECOG PS ≥ 2), Normal LDH (for Elevated LDH), ALK-positive ALCL (for non-ALK-positive PTCL), and BV plus CHP-like (for CHOEP).

Abbreviations: OS, overall survival; PFS, progression-free survival; HR, hazard ratio; CI, Confidence Interval; ENSs, extranodal sites; BMI, bone marrow infiltration; ECOG PS, Eastern Cooperative Oncology Group performance status; LDH, lactate dehydrogenase; ALCL, anaplastic large cell lymphoma; PTCL, peripheral T-cell lymphoma.

Table S3

Multivariable analysis for the overall survival and progression-free survival.

Prognostic factors	OS		PFS	
	**HR (95%CI)**	**Cox P-value**	**HR (95%CI)**	**Cox P-value**
BMI (yes vs. no)	4.069 (1.638-10.110)	**0.003**	2.432 (1.232-4.800)	**0.010**
Elevated LDH (yes vs. no)	2.997 (1.133-7.925)	**0.027**	2.656 (1.349-5.226)	**0.005**
Subtype (non-ALK+ PTCL vs. ALK+ ALCL)	Not included*	-	4.322 (1.663-11.233)	**0.003**
Treatment (CHOEP vs. BV plus CHP-like)	Not included*	-	1.865 (0.959-3.624)	0.066

Note: *, Subtype and treatment were not included in the OS multivariable model due to complete separation.

HR >1 indicates an increased risk of death (OS) or progression/death (PFS). Reference categories: BMI no; LDH not elevated; ALK+ ALCL; BV plus CHP-like.

Multivariable models were constructed according to the Events Per Variable (EPV) principle. For OS, only 19 events occurred, allowing a maximum of 2 covariates; BMI and elevated LDH were included based on their significance in univariable analysis. For PFS, 40 events occurred, allowing a maximum of 4 covariates; BMI, elevated LDH, subtype, and treatment were included based on their significance in univariable analysis ($P < 0.05$).

Abbreviations: OS, overall survival; PFS, progression-free survival; HR, hazard ratio; CI, Confidence Interval; BMI, bone marrow infiltration; LDH, lactate dehydrogenase; ALCL, anaplastic large cell lymphoma; PTCL, peripheral T-cell lymphoma.
